# Cerebellar Involvement in an Immunocompetent Patient Presenting with Progressive Multifocal Leukoencephalopathy

**DOI:** 10.1155/2017/2396068

**Published:** 2017-08-24

**Authors:** Rafael Garcia-Carretero, Blanca San Jose Montano

**Affiliations:** ^1^Department of Internal Medicine, Mostoles University Hospital, Madrid, Spain; ^2^Health Science Library, Mostoles University Hospital, Madrid, Spain

## Abstract

Progressive multifocal leukoencephalopathy (PML) is a demyelinating disease caused by the JC virus, a polyomavirus that can be reactivated under certain immunosuppressive conditions, such as AIDS, immunomodulatory therapy, and haematological malignancies. However, a few cases of immunocompetent patients have been reported in which no immunodeficiency was present. We describe the case of an 83-year-old immunocompetent man who presented with severe cerebellar symptoms with an MRI scan suggestive of severe demyelinating disease. We were not able to identify any occult immunosuppression or malignancy in our patient.

## 1. Introduction

The JC virus is a ubiquitous polyomavirus infecting 50–80% of humans, known because it causes progressive multifocal encephalopathy (PML) in patients with AIDS and other immunosuppressed patients [1].*⁠* JC virus infection is acquired during adolescence but is usually asymptomatic. After this primary infection, it remains latent in the kidney and lymphoid tissue but can be reactivated under a certain degree of immunosuppression [1].*⁠* PML is very rare in immunocompetent patients, and therefore clinicians must search for any occult underlying disorders.

## 2. Case Presentation

An 83-year-old male patient was admitted to the hospital presenting with nausea, unsteadiness, and difficulties in walking. He had a two-month history of falls and abnormal gait. He had atrial fibrillation and was on acenocumarol (a vitamin K antagonist anticoagulant), and his family was concerned because of the risk of major bleeding after a fall. On physical examination, he had unsteady gait and dysdiadochokinesia. Eye-hand and eye-knee tests were abnormal. Cranial nerves and speech were normal, and the rest of the examination was unremarkable.

Anticoagulant therapy was discontinued due to the risk of a fall and major bleeding.

Routine laboratory data were normal, including kidney and liver panels. Haemoglobin, white blood cell count, and platelets were normal. A CT scan of the brain was performed, which did not show any acute abnormalities.

Over the days that followed, a gradual worsening occurred, with dysarthria, absent gag reflex and dysphagia, poor attention span, generalised hyperreflexia, and vertical nystagmus.

**Figure 1:**
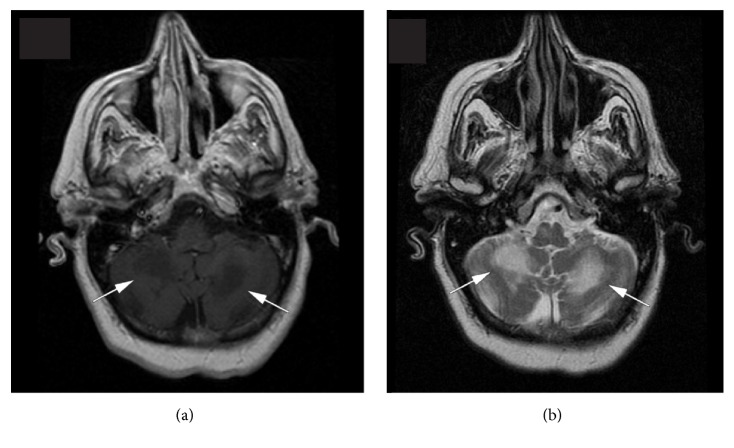
While the brain parenchyma was normal, the MRI shows asymmetric hypointense lesions in both cerebellar hemispheres on T1-weighted images (Slide (a)) and hyperintense lesions on T2-weighted images (Slide (b)).

An MRI of the brain showed an asymmetric abnormal signal in both cerebellar peduncles, the cerebellar white matter, and the front area of the medulla (Figure 1). These abnormalities were hypointense on T1-weighted images and hyperintense on T2-weighted images, with no gadolinium enhancement and no diffusion restriction. The right side structures were the most affected. The rest of the brain parenchyma was normal.

## 3. Investigations

We consulted our neurologist to establish a differential diagnosis-based work-up. The abnormalities were not likely to be ischaemic or cancerous. However, inflammatory conditions, infectious diseases, primary central nervous system lymphoma, paraneoplastic neurologic syndrome, and demyelinating diseases should be ruled out.

Serologic tests including toxoplasma, HIV, CMV, and herpes simplex were negative. Immunological tests such as C3 and C4, immunoglobulin levels, and lymphocyte subsets were also normal. Tumour markers CA19.9, CEA, CA125, and PSA were normal. An abdominal ultrasound and a thoracic-abdominal CT scan did not reveal any abnormalities. A lumbar puncture was performed and the cerebrospinal fluid (CSF) revealed a glucose level of 110 mg/dl, proteins 42 mg/dl (normal range: 15–45 mg/dl), and leucocytes 1/*μ*l. No oligoclonal bands were found.

## 4. Outcome and Follow-Up

While performing the work-up, two weeks after admission, the patient's condition deteriorated, with worsening of the neurological symptoms. He was lethargic and stuporous, with pauses in breathing. He started on steroid pulse therapy (1 g methylprednisolone daily for 5 days), but with no improvement.

A sample of the CSF was sent to a specialised laboratory in order to assess antineuronal antibodies (anti-Hu, anti-Yo, and anti-Ri), which were negative. PCR for neurotropic viruses were also performed, and the JC virus PCR was positive in the CSF. The finding of the JC virus was reported the same day the patient passed away, three weeks after admission.

## 5. Discussion

Progressive multifocal leukoencephalopathy (PML) is a demyelinating disease caused by the JC virus and involves the cerebral white matter. JC virus reactivation is considered an opportunistic infection in AIDS patients or in situations of severe immunosuppression, such as malignancies, haematological and lymphoproliferative disorders, or monoclonal antibody therapy (natalizumab and alemtuzumab) [2, 3].*⁠* In AIDS patients, the prevalence of PML is 5%, which is decreasing, thanks to antiretroviral therapy. PML is also linked to sarcoidosis, and it is not not associated with immunosuppression or CD4 lymphocytopenia [4].

Our patient did not have any apparent underlying disorder, occult malignancy, or immunosuppressive treatment. Several case reports have been published on patients without an immunocompromised status [5].*⁠* In this paper, a certain degree of immunosuppression was reported, such as liver cirrhosis and other systemic diseases. Therefore, severe immunosuppression would not be a prerequisite for PML.

The work-up, however, should lead clinicians to identify the underlying cause, in an attempt to find any occult, undetected immunosuppressive condition. A recently published paper [6]*⁠* reports a specific defect in the interferon gamma production in an apparently immunocompetent patient presenting PML. Therefore, in addition to the routine immunological screening, this paper proposes that patients suspected to have PML should be tested for interferon gamma deficiency.

PML affects the white matter of the cerebral hemispheres, medulla, and cerebellum, and the clinical symptoms depend on the area involved: confusion, dizziness, behavioural changes, hyperreflexia, ataxia, and so forth. Cognitive deficits are typical clinical manifestations in PML, due to the involvement of the subcortical hemispheric regions. Infratentorial, brain stem and cerebellar manifestations are rare. However, not only some cases of immunocompromised patients but also AIDS patients under highly active antiretroviral therapy or under rapid immune reconstitution, that is, with an improved immune status, with PML affecting the posterior fossa have been reported [7–12].*⁠* We depict a new case of a purely cerebellar PML in an immunocompetent patient.

Clinical course and neuroimaging findings should lead us to include PML in the differential diagnosis list. Although the JC virus can be detected by PCR in CSF, the definitive diagnosis should be made by detecting JC virus DNA or antigens in a brain biopsy.

There is no specific antiviral treatment. The damage caused by PML cannot be reversed, as the areas involved are unable to remyelinate. Patients will therefore present chronic neurological sequelae. Patients may remain stable after discontinuing the immunosuppressive treatment, after optimisation of antiretroviral therapy in AIDS, or after specific treatment for immune system recovery. Although several regimes have been applied (mirtazapine and oral antimalarial drugs) [13],*⁠* the results are inconclusive. However, Christakis et al. [14] reported the case of an immunocompetent patient with a favourable outcome with mirtazapine and mefloquine, whose neurological symptoms stabilised and neuroimaging even improved. In our patient, supportive care and steroid pulse therapy were not useful and the clinical course was rapidly progressive and fatal.

According to the PML diagnostic criteria [15],*⁠* we could establish a diagnosis of probable PML, based on three features: presence of JC virus PCR positivity in CSF and compatible MRI findings, but atypical clinical presentation. Although we cannot consider a definitive diagnosis of PML, patients with probable PML should be managed as PML.

## 6. Learning Points

- The JC virus is a ubiquitous pathogen that can reactivate under certain immunosuppressive disorders, such as AIDS, malignancies, and haematological diseases, but also in aggressive monoclonal antibody therapies.
- Clinicians should rule out PML in immunocompromised status but also in immunocompetent patients, because occult or minimal predisposing conditions can be present.
- Clinical course, neuroimaging, and findings in CSF are highly suggestive of PML, but the definitive diagnosis should be confirmed by detecting DNA or antigens in a brain biopsy.
- There is no specific antiviral treatment, and some drugs, such as mirtazapine and mefloquine, have shown inconclusive results.
